# Proteorhodopsin Phototrophy in Antarctic Coastal Waters

**DOI:** 10.1128/mSphere.00525-21

**Published:** 2021-08-18

**Authors:** Jerónimo Cifuentes-Anticevic, María E. Alcamán-Arias, Tomás Alarcón-Schumacher, Javier Tamayo-Leiva, Carlos Pedrós-Alió, Laura Farías, Beatriz Díez

**Affiliations:** a Department of Molecular Genetics and Microbiology, Pontificia Universidad Católica de Chile, Santiago, Chile; b Department of Oceanography, Universidad de Concepción, Concepción, Chile; c Center for Climate and Resilience Research (CR)^2^, Santiago, Chile; d Escuela de Medicina, Universidad Espíritu Santo, Samborondon, Ecuador; e Max Planck Institute for Marine Microbiology, Bremen, Germany; f Departamento de Biología de Sistemas, Centro Nacional de Biotecnología (CSIC), Madrid, Spain; g Center for Genome Regulation (CRG), Santiago, Chile; Clemson University

**Keywords:** Antarctica, photoheterotrophy, proteorhodopsin, marine microbiology, metagenomics, metatranscriptomes, sunlight

## Abstract

Microbial proton-pumping rhodopsins are considered the simplest strategy among phototrophs to conserve energy from light. Proteorhodopsins are the most studied rhodopsins thus far because of their ubiquitous presence in the ocean, except in Antarctica, where they remain understudied. We analyzed proteorhodopsin abundance and transcriptional activity in the Western Antarctic coastal seawaters. Combining quantitative PCR (qPCR) and metagenomics, the relative abundance of proteorhodopsin-bearing bacteria accounted on average for 17, 3.5, and 29.7% of the bacterial community in Chile Bay (South Shetland Islands) during 2014, 2016, and 2017 summer-autumn, respectively. The abundance of proteorhodopsin-bearing bacteria changed in relation to environmental conditions such as chlorophyll *a* and temperature. *Alphaproteobacteria*, *Gammaproteobacteria*, and *Flavobacteriia* were the main bacteria that transcribed the proteorhodopsin gene during day and night. Although green light-absorbing proteorhodopsin genes were more abundant than blue-absorbing ones, the latter were transcribed more intensely, resulting in >50% of the proteorhodopsin transcripts during the day and night. *Flavobacteriia* were the most abundant proteorhodopsin-bearing bacteria in the metagenomes; however, *Alphaproteobacteria* and *Gammaproteobacteria* were more represented in the metatranscriptomes, with qPCR quantification suggesting the dominance of the active SAR11 clade. Our results show that proteorhodopsin-bearing bacteria are prevalent in Antarctic coastal waters in late austral summer and early autumn, and their ecological relevance needs to be elucidated to better understand how sunlight energy is used in this marine ecosystem.

**IMPORTANCE** Proteorhodopsin-bearing microorganisms in the Southern Ocean have been overlooked since their discovery in 2000. The present study identify taxonomy and quantify the relative abundance of proteorhodopsin-bearing bacteria and proteorhodopsin gene transcription in the West Antarctic Peninsula’s coastal waters. This information is crucial to understand better how sunlight enters this marine environment through alternative ways unrelated to chlorophyll-based strategies. The relative abundance of proteorhodopsin-bearing bacteria seems to be related to environmental parameters (e.g., chlorophyll *a*, temperature) that change yearly at the coastal water of the West Antarctic Peninsula during the austral late summers and early autumns. Proteorhodopsin-bearing bacteria from Antarctic coastal waters are potentially able to exploit both the green and blue spectrum of sunlight and are a prevalent group during the summer in this polar environment.

## INTRODUCTION

Seasonal light availability during the Antarctic summer plays a critical role in shaping phytoplankton and bacterioplankton communities, which are central players in the biogeochemical cycles and food webs of marine ecosystems such as the Southern Ocean (1, 2). Seasonal variation in sea ice cover and day length modulates light availability, resulting in high productivity in the summer and very low productivity in the winter (3–5). During the spring/summer, photoautotrophs (such as diatoms and haptophytes) use light as a primary energy source via chlorophyll *a* (Chl*a*) (6). However, photoheterotrophic microorganisms possess two additional mechanisms for harvesting sunlight energy: (i) bacteriochlorophyll-based photosystems (7) and (ii) rhodopsin (8, 9). Aerobic anoxygenic phototrophic bacteria use bacteriochlorophyll *a* and several other pigments (i.e., carotenoids) to capture light, as well as sophisticated machinery to transport protons across the membrane (10). In contrast, rhodopsin-based phototrophy, the simplest strategy among phototrophs, consists of a single integral membrane protein with a covalently bonded retinal (11). To date, proteorhodopsin is the most studied rhodopsin because of its presence in different bacterial phyla and its wide distribution in the ocean (12). However, there are few quantitative measurements of proteorhodopsin-bearing bacteria available for marine environments, where it is been suggested that proteorhodopsins are a major energy-conserving strategy to capture sunlight in the surface ocean (13). Particularly, in high latitude environments, which exhibit marked changes in light variability, the presence and abundance of proteorhodopsin-bearing bacteria have been documented in both the Beaufort and Chukchi Seas of the Arctic (14–16). For example, proteorhodopsin-bearing bacteria, mainly affiliated with *Alphaproteobacteria*, accounted for 1% to 45% of the marine bacterial abundance throughout the photic zone during the summer in the Beaufort Sea (16), and proteorhodopsin gene transcripts have been found during both the winter and summer seasons (15).

So far, the presence and expression of proteorhodopsin genes in the Antarctic marine ecosystem remain much more understudied than in the Arctic Ocean (see Table 1 for the limited references of proteorhodopsin genes in the Antarctic marine ecosystem). The first report of a functional blue-absorbing proteorhodopsin (blue-PR) in Antarctica was reported in 2001 at Palmer Station (Anvers Island) on the west coast of the Antarctic Peninsula (8). Subsequently, transcription of proteorhodopsin genes related to *Flavobacteriia* (*Polaribacte*r), *Alphaproteobacteria* (SAR11 clade), and *Gammaproteobacteria* (SAR92 clade) in the sea ice microbial community from the Ross Sea region during the austral summer was reported (17). The presence of the gene and protein was demonstrated through metagenomics and metaproteomics in the coastal surface seawater of Palmer Station (18, 19). Finally, concentrations of rhodopsin-based photosystems were determined using retinal as a proxy in the subantarctic waters of the Subtropical Frontal Zone off New Zealand, showing that the abundance of rhodopsin-based photosystems was, on average, 20 times higher than that of Chl*a*-based photosystems (20). However, in Antarctica’s coastal waters, the relative contribution of proteorhodopsin-bearing bacteria to the microbial community remains elusive, as the effect of environmental parameters on the transcription of the proteorhodopsin gene. Thus, the present study is the first to identify the taxonomic affiliation and relative abundance of proteorhodopsin-bearing bacteria, as well as proteorhodopsin gene transcription, in coastal marine waters of the West Antarctic Peninsula (WAP) under contrasting environmental conditions (Chl*a* levels) and light availability (during day and night). The information provided here will help to understand how proteorhodopsin-driven phototrophy contributes to enter sunlight energy into this ecosystem. Additionally, we investigated whether proteorhodopsin can act as a light-driven proton pump by analyzing the transcript levels of enzymes involved in the retinal biosynthetic pathway and determining ion pumping and spectral tuning residues of proteorhodopsin protein sequences.

**TABLE 1:** Proteorhodopsin sequences identified in Antarctic marine samples and main findings regarding taxonomy and absorption spectra (July 2021)

Sampling site	Finding(s)	Absorbing spectra	Taxon^*a*^	Methodology	Reference
Palmer Station	Proteorhodopsin gene present	Blue		Fosmid clone	8
Palmer Station	Proteorhodopsin gene present	Blue	Likely *Proteobacteria*	Fosmid clone	25
Ross Sea sea ice	Proteorhodopsin gene and transcript in sea ice bacteria	Blue and green	*Alphaproteobacteria*, *Gammaproteobacteria*, and *Bacteroidetes*	PCR amplicons	17
Palmer Station	Proteorhodopsin present in summer and winter		SAR11, OMG	Metaproteome	19
Palmer Station	Proteorhodopsin present in summer and winter			Metagenome	18
King George Island Potter Cove sediments	Proteorhodopsin in viruses and bacteria	Green	Many	Metagenome	26
King George Island	Proteorhodopsin gene present	Blue and green		PCR amplicons	27
Subtropical frontal zone off New Zealand (subantarctic waters)	Rhodopsin photosystem concn			Quantification of retinal as proxy a for rhodopsins	20

a Entries in the Taxon column correspond to the reported proteorhodopsin-bearing microorganisms in the original reference. OMG, oligotrophic marine *Gammaproteobacteria* group.

We investigated proteorhodopsin-bearing bacterial dynamics across three austral late summers-early autumns (2014, 2016, and 2017) in Chile Bay (South Shetland Islands, WAP). The shallow water (∼200 m) of Chile Bay is subjected to both strong intraseasonal and interannual variability, mainly due to tidal and wind-driven processes (21), with significant modulation by climate events like El Niño-Southern Oscillation and the Southern Annular Mode (22). Finally, Antarctic surface water (23), which is warmer (>0°C), fresher (salinity <33.5), and richer in nutrients (nitrate >15 μM), is carried to Chile Bay by the Bransfield Current.

## RESULTS

### Environmental conditions at the study site.

**FIG 1:**
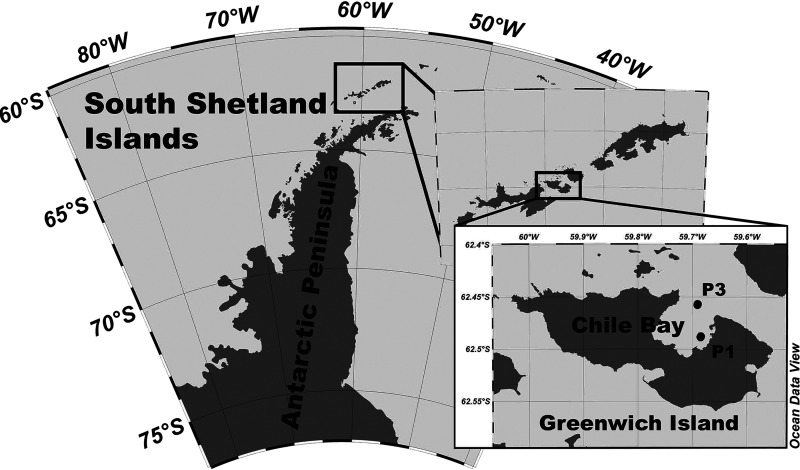
Map of Chile Bay on Greenwich Island, South Shetland Islands, Antarctica. Sampling sites P1 (62.49^o^S, 59.66^o^W) and P3 (62.49^o^S, 59.66^o^W) are indicated by black circles on the inset map.

Marine surface (2 m) and subsurface (30 m) waters of the two locations in Chile Bay (Fig. 1) were monitored between February and March during the summers/autumns of 2014 (*n* = 4), 2016 (*n* = 10), and 2017 (*n* = 19). Environmental data from 2014, 2016, and 2017 summers are summarized in Table 2 (metadata for each sample are shown in Table S1 in the supplemental material), and data from 2014 are also published (24, 25). Briefly, in 2014, surface water temperature and Chl*a* increased from −0.1°C and 0.3 mg m^−3^ in February to 0.3°C and 2.5 mg m^−3^ in March (24, 25). In 2016, Chl*a* concentration varied from 1.0 to 11.4 mg m^−3^, and seawater temperature varied from −0.2 to 0.8°C. In 2017, the Chl*a* levels were between 0.2 and 1.29 mg m^−3^, and the seawater temperature was the highest of the 3 years (1.4 to 2.7°C). Nutrients such as nitrate and phosphate levels were on average >15.56 μM and >1.35 μM, respectively, for all years (Table 2).

**TABLE 2:** Environmental variables obtained in Chile Bay during the summers of 2014, 2016, and 2017^*a*^

Year	Date (day.month)	Sampling point	Depth| (m)	Time	Temp (°C)	Salinity (PSU)	Nitrate (μM)	Nitrite (μM)	Ammonium (μM)	Phosphate (μM)	Silicate (μM)	Chlorophyll (mg m^−3^)	Analysis	*n*
2014	11.02	P3	2	N	−0.1^*b*^	33.1^*b*^	22.4	0.17	nd	1.86	46.8	0.31	MetaT	1
	14.02	P3	2	D	−0.1^*b*^	33.2^*b*^	19.5	0.16	nd	1.61	43.6	0.36	MetaG-MetaT	1
	03.03	P3	2	D	0.3^*b*^	33.5^*b*^	20.2	0.16	nd	1.59	44.1	2.38	MetaT	1
	04.03	P3	2	N	0.3^*b*^	33.9^*b*^	17.7	0.10	nd	1.28	37.9	2.53	MetaG-MetaT	1
2016	18.02−04.03	P1	2	D/N	0.1 ± 0.3	nd	15.56 ± 2.95	0.29 ± 0.03	nd	1.35 ± 0.26	51.57 ± 8.71	5.8 ± 4.3	(RT-)qPCR	4
		P3	2	D/N	−0.16 ± 1.14	nd	16.34 ± 3.09	0.28 ± 0.04	nd	1.46 ± 0.23	51.28 ± 7.42	1.97 ± 0.98	(RT-)qPCR	6
2017	08.02−25.02	P1	2	D/N	2.06 ± 0.41	33.89 ± 0.19	20.09 ± 3.01	0.19 ± 0.02	0.67 ± 0.24	1.43 ± 0.13	49.79 ± 7.98	0.59 ± 0.31	(RT-)qPCR	5
		P3	2	D/N	2.03 ± 0.32	33.95 ± 0.12	22.56 ± 2.11	0.21 ± 0.02	0.56 ± 0.19	1.54 ± 0.12	50.13 ± 8.06	0.5 ± 0.34	(RT-)qPCR	9
		P3	30	D/N	1.53 ± 0.12	34.16 ± 0.01	23.41 ± 4.55	0.20 ± 0.03	0.66 ± 0.14	1.62 ± 0.13	53.24 ± 6.43	0.33 ± 0.17	qPCR	5

a Data are means ± standard deviations. Abbreviations: D, daytime; N, nighttime; PSU, practical salinity unit; nd, no data; Meta-G, metagenome; Meta-T, metatranscriptome; (RT-)qPCR, PR gene and transcript quantification by qPCR; qPCR, PR gene quantification by qPCR.

b During 2014, temperature and salinity were measured using a multiparameter sensor (Oakton PCD650).

10.1128/mSphere.00525-21.4 TABLE S1 Metadata for each sample from summers 2016 and 2017. Download Table S1, XLSX file, 0.01 MB. Copyright © 2021 Cifuentes-Anticevic et al. 2021 Cifuentes-Anticevic et al. https://creativecommons.org/licenses/by/4.0/ This content is distributed under the terms of the Creative Commons Attribution 4.0 International license.

### Abundance, phylogenetic analysis, and daily transcriptional activity of proteorhodopsin genes.

**FIG 2:**
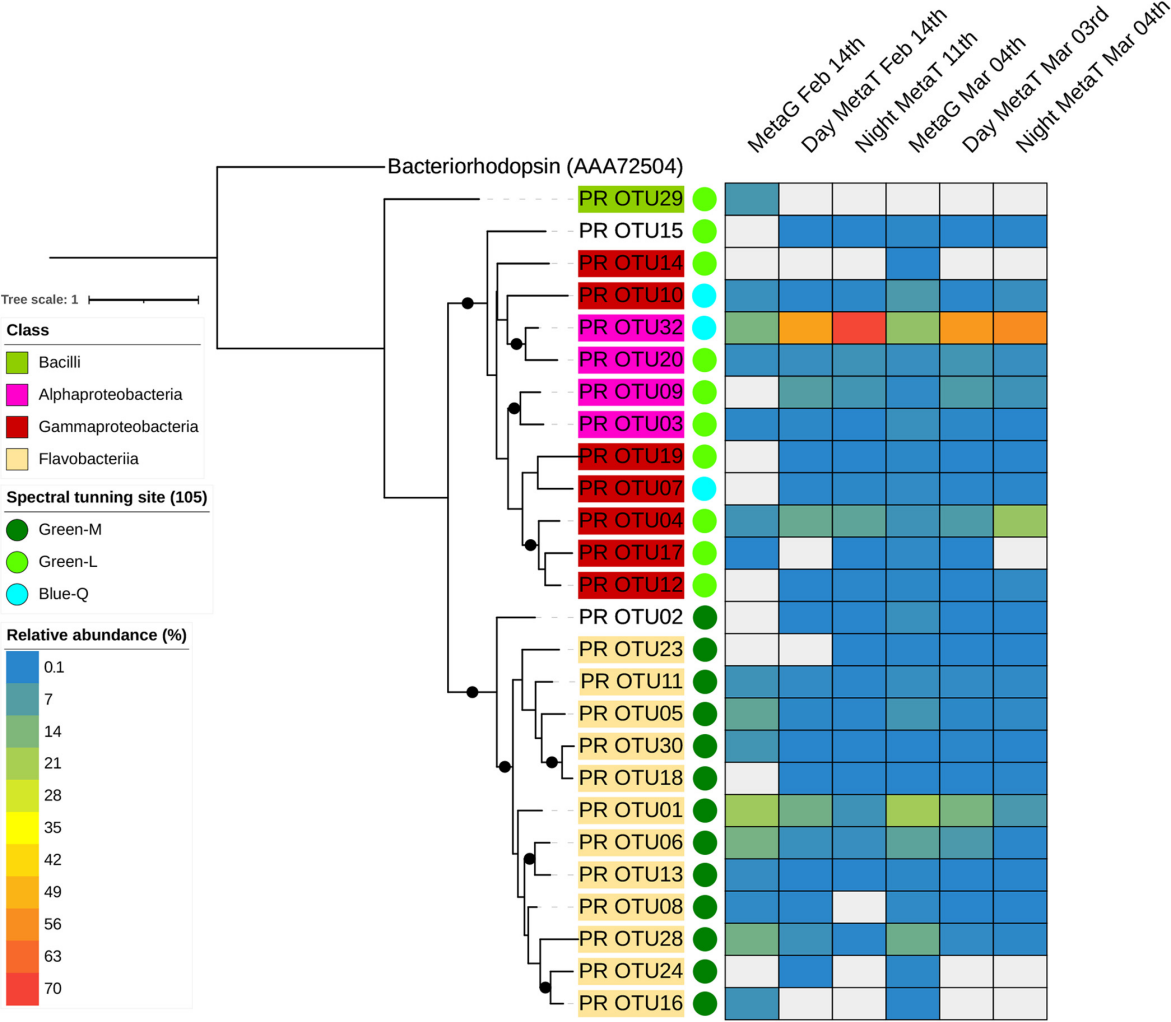
Phylogenetic reconstruction of proteorhodopsin proteins (PR OTUXX) and their relative abundance in metagenomes (MetaG) and metatranscriptomes (MetaT) from austral summer 2014 in Chile Bay. The color scale (blue-yellow-red) represents those sequences with a relative abundance from >0.1% to 70%, while light gray represents those sequences with a relative abundance from 0 to 0.1%. Taxonomic classification was inferred from the phylogenetic placement of proteorhodopsin sequences from Chile Bay in the phylogenetic reconstruction with reference sequences (see Fig. S1 in the supplemental material). Taxonomy of the reference sequences was obtained from metadata available in the MicRhoDE database (56). The tree is rooted to Halobacterium salinarum bacteriorhodopsin (NCBI accession no. AAA72504). Black circles above the nodes indicate >95% Ultra Fast bootstrap support and >80% SH-alrt branch support.

**FIG 3:**
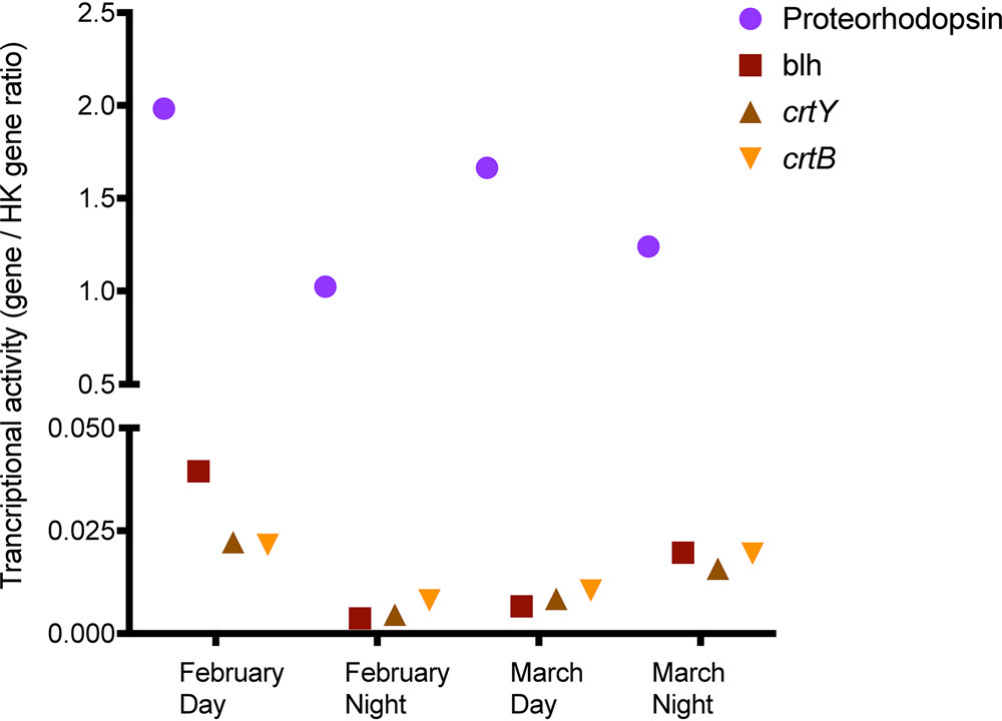
Relative transcriptional levels of proteorhodopsin gene and retinal biosynthetic genes during the austral summer of 2014 under low (February) and high (March) chlorophyll *a* concentrations in the seawater. Relative transcript abundances were calculated using the ratio of transcripts for each gene: proteorhodopsin, β-carotene dioxygenase (*blh*), lycopene cyclase (*crtY*), and phytoene synthase (*crtB*) to the mean transcript abundance of the housekeeping (HK) genes (*rplB*, *rpoB*, and EF-Tu).

The proteorhodopsin gene abundance, taxonomic affiliation, and transcriptional expression (day and night) were investigated on two metagenomes and four metatranscriptomes (two daytime, two nighttime) from different sampling periods of the summer of 2014 (24). The metagenomic analysis revealed 66 proteorhodopsin gene sequences. Comparing the abundance of proteorhodopsin reads to single-copy genes, we determined that proteorhodopsin-bearing bacteria represented on average 17% ± 4% and 17% ± 7%, in February and March, respectively, of the total bacteria. The results from meta-omic analyses are shown in Fig. 2, including the relative abundance of each proteorhodopsin operational taxonomic unit (OTU) during the two periods (February and March) and their transcription during the day and night. The taxonomic composition of the proteorhodopsin sequences was very similar for February and March (Fig. 2; see also Fig. S1 in the supplemental material). Proteorhodopsin reads in both metagenomes were mainly affiliated with *Bacteroidetes* (70 to 55%; primarily *Flavobacteriia*), *Alphaproteobacteria* (17 to 25%; SAR11 and SAR116), and *Gammaproteobacteria* (8 to 16%; SAR86 and SAR92) (Fig. S1 and Table S2). Conversely, the metatranscriptomic reads for all samples showed that *Alphaproteobacteria* accounted for a larger fraction than *Flavobacteriia*, for which all members were underrepresented in the metatranscriptomes. A blue-PR from SAR11 clade was the most transcribed proteorhodopsin gene, while a green-absorbing proteorhodopsin (green-PR) from this same clade was transcribed to a lesser extent. *Gammaproteobacteria* were also proportionally more abundant in the metatranscriptomes than in the metagenomes. SAR92 and SAR86 were the main *Gammaproteobacteria* that transcribed the proteorhodopsin gene, while PR OTU01 and PR OTU06 were the main proteorhodopsin transcribed genes among the *Flavobacteriia*. The differences found between night and day may be attributed to the fact that transcription was generally higher in daytime, depicting that light availability may regulate proteorhodopsin gene transcription differently for every taxa (Fig. 3). It appears that *Flavobacteriia* transcription was considerably reduced at night, resulting in a larger proportion of the expression being attributed to “*Candidatus* Pelagibacter.” Furthermore, in searching for retinal biosynthetic enzymes, we found gene sequences for *blh*, *crtY*, and *crtB* in both metagenomes, and they were transcribed during both the day and night (Fig. 3). Thus, proteorhodopsin-bearing bacteria should be able to synthesize retinal or acquire it from the environment and use it as a chromophore. However, retinal biosynthetic enzymes were considerably less transcribed than the proteorhodopsin gene (>10-fold), with no observed difference between day and nighttime samples.

10.1128/mSphere.00525-21.1 FIG S1 Phylogenetic reconstruction of proteorhodopsin proteins (PR OTUXX in bold) obtained from the coastal water metagenomes during the austral summer of 2014 in Chile Bay. The taxonomy (class) of the reference sequences was obtained from metadata available in the MicRhoDE database. Green (methionine [dark green] or leucine [light green]) and cyan (glutamine) circles indicate spectral turning amino acids. Proteorhodopsin sequences retrieved from other studies in polar regions where placed phylogenetically on the reference tree, and their polar origin is indicated by dark blue (Antarctica) or red (Arctic) circles. The tree is rooted to Halobacterium salinarum rhodopsin (AAA72504). Black circles over nodes indicate ultrafast bootstrap support of >95% and SH-alrt branch support of >80%. Download FIG S1, PDF file, 1.5 MB. Copyright © 2021 Cifuentes-Anticevic et al. 2021 Cifuentes-Anticevic et al. https://creativecommons.org/licenses/by/4.0/ This content is distributed under the terms of the Creative Commons Attribution 4.0 International license.

10.1128/mSphere.00525-21.5 TABLE S2 Relative abundance of PR genes by OTU and color in the metagenomes and metatranscriptomes of Chile Bay. The relative abundance of each representative PR gene was corrected for gene size. The taxonomic affiliation of PR OTUs is related to the phylogeny obtained in Fig. S1. Download Table S2, XLSX file, 0.01 MB. Copyright © 2021 Cifuentes-Anticevic et al. 2021 Cifuentes-Anticevic et al. https://creativecommons.org/licenses/by/4.0/ This content is distributed under the terms of the Creative Commons Attribution 4.0 International license.

### Proteorhodopsin functional domain.

**FIG 4:**
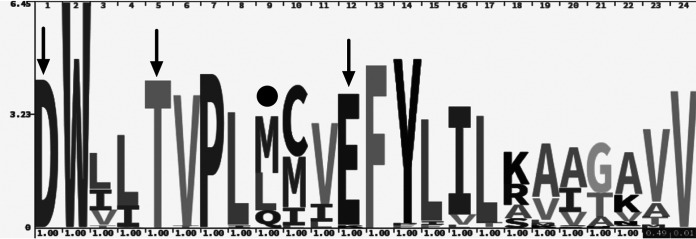
HMM logo of the proteorhodopsin proton pumping domain and spectral tuning amino acid retrieved from Chile Bay. The arrows indicate amino acids involved in proton pumping at positions 97, 101, and 108. The solid black circle indicates the amino acid of the spectral tuning position at position 105.

All retrieved proteorhodopsin sequences harbored the most conserved proton pumping domain (aspartic acid, threonine, and glutamic acid at positions 97, 101, and 108, respectively), except for one sequence from *Exiguobacterium*, which had a lysine at position 108. Thus, all of the proteorhodopsin genes encoded a fully functional protein that, in turn, could act as a light-driven proton pump (Fig. 4). In the light tuning position (position 105), proteorhodopsin sequences mostly harbored leucine or methionine (common in green-PR), while some had glutamine (typical in blue-PRs). During both months, green-PR genes (83 to 73% in February and March, respectively) were more abundant than blue-PR genes (Fig. 2 and Table S2). However, the transcription of green-PR was comparable to that of blue-PR during the day and night, were blue-PR accounted for more than 50% of the proteorhodopsin transcripts during day and night (Table S2). Throughout the summer of 2014, SAR11 was the main taxon transcribing blue-PR, while other proteorhodopsin-bearing bacteria related to the *Flavobacteriia* class SAR92 and SAR86 clade transcribed green-PR (Fig. 2 and Table S2).

### Daily temporal variability of the proteorhodopsin gene abundance and transcriptional activity.

**FIG 5:**
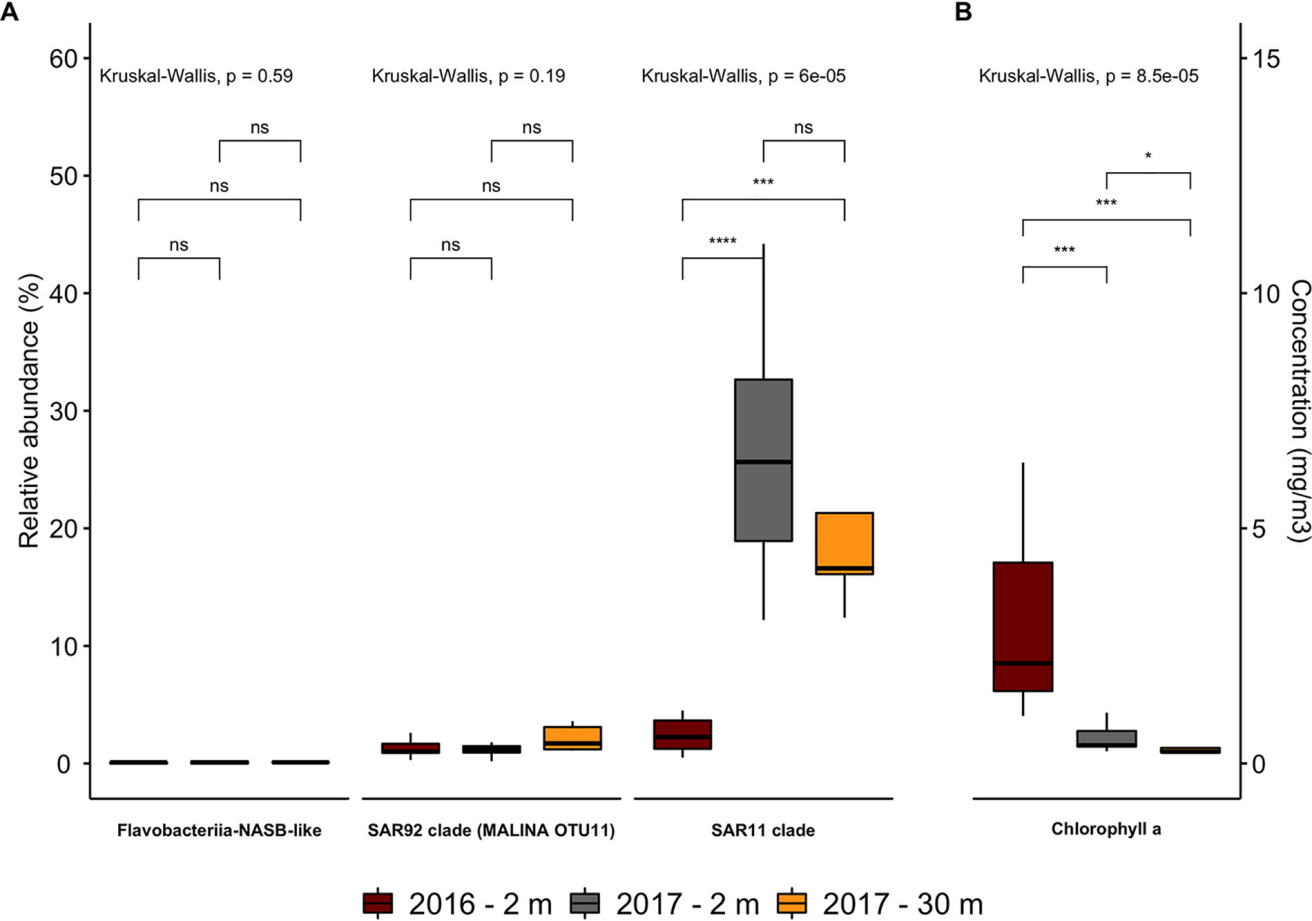
(A and B) Relative abundance and variability of proteorhodopsin-bearing bacteria (A) and chlorophyll *a* levels in Chile Bay during the austral summers of 2016 and 2017 (B). The abundance of proteorhoodpsin-bearing bacteria was quantified using DNA samples from the late summer of 2016 and 2017. Analyses of variance between taxonomic groups are shown by date (i.e., 2016 versus 2017) and depth (i.e., 2 m versus 30 m). *P* values from the Kruskal-Wallis test are shown by group comparison. Pairwise comparisons between groups are shown by Wilcoxon tests, with statistical significance indicated as follows: ns, not significant; *, *P* ≤ 0.05; ***, *P* ≤ 0.001; ****, *P* ≤ 0.0001 (for 2016, 2 m, *n* = 10; for 2017, 2 m, *n* = 14; 30 m, *n* = 5).

An estimation of the relative abundance of proteorhodopsin-bearing bacteria in Chile Bay was determined by quantitative PCR (qPCR) for samples taken at 2 m and 30 m during 2016 (*n* = 10) and 2017 (*n* = 19) at the two different sampling sites (Fig. 1). We did not find any significant differences (Kruskal-Wallis [KW], *P* > 0.5) in the relative abundances of proteorhodopsin-bearing bacteria from the targeted taxa between the two sampling sites, therefore both points were considered replicates (Tables S3 and S4). Through qPCR, we estimated that the relative abundance of proteorhodopsin-bearing bacteria from the SAR11 clade, SAR92 clade, and *Flavobacteria* from the NASB clade (Flavo-NASB-like) ranged from 1.1 to 5.7% of the bacterial community in 2016, whereas a higher relative abundance was found in 2017 (12.6 to 63.3%). In both years, proteorhodopsin sequences of the SAR11 clade were more abundant than those of the SAR92 clade and Flavo-NASB-like (Table S4 and Fig. S2). Additionally, SAR11 proteorhodopsin sequences were more abundant in 2017 (at both depths) than in 2016 (KW, *P* = 6e−5 [Fig. 5A]), while no significant differences between years and depths were found for the SAR92 clade or the Flavo-NASB-like clade (Table S4 and Fig. 5A). When comparing the relative abundances of SAR11 proteorhodopsin genes between the 2 years, it was higher during 2017, inversely to the Chl*a* levels from Chile Bay, which were higher during 2016 (KW, *P* = 8.5e−5; Fig. 5B). Spearman’s correlation (80) was used to estimate correlations between physicochemical and biological data. Environmental variables, such as temperature, Chl*a*, nitrite, nitrate, and phosphate, correlated with the relative abundances of proteorhodopsin genes from the SAR11 clades (Fig. S3). Temperature (*r* = 0.64, *P* < 0.05), ammonia (*r* = 0.73, *P* < 0.05), and nitrate (*r* = 0.48, *P* < 0.05) correlated positively with the SAR11 proteorhodopsin gene abundance, while Chl*a* levels (*r* = −0.51, *P* < 0.05) and nitrite (*r* = −0.71, *P* < 0.05) correlated negatively with this taxon. The relative abundance of the Flavo-NASB-like proteorhodopsin gene positively correlated with ammonia levels (*r* = 0.57, *P* < 0.05). Other environmental variables, including salinity, oxygen (both available only for 2017), silicate, and phosphate, did not significantly correlate with any proteorhodopsin gene abundances (Fig. S3). A permutational multivariate analysis of variance (PERMANOVA) showed that there were significant differences in the relative abundances of the proteorhodopsin gene from SAR11 and SAR92 clades in the marine microbial community with temperature (PERMANOVA, SAR11 *R*^2^ = 0.418, *P* < 0.01) and depth (PERMANOVA, SAR92 *R*^2^ = 0237, *P* < 0.05), respectively (Table S5).

10.1128/mSphere.00525-21.2 FIG S2 Variability of proteorhodopsin-bearing bacteria; abundances among taxonomic groups, year, and depth. Analyses of variance between taxonomic groups are shown by date (i.e., 2016 versus 2017) and depth (i.e., 2 m versus 30 m). *P* values from the Kruskal-Wallis test are shown by group comparison. Pairwise comparisons between groups are shown by Wilcoxon tests, with significance indicated as follows: ns, not significant; **, *P* ≤ 0.01; ***, *P* ≤ 0.001; ****, *P* ≤ 0.0001 (for 2016, 2 m, *n* = 10; for 2017, 2 m, *n* = 14; 30 m, *n* = 5). Download FIG S2, PDF file, 0.3 MB. Copyright © 2021 Cifuentes-Anticevic et al. 2021 Cifuentes-Anticevic et al. https://creativecommons.org/licenses/by/4.0/ This content is distributed under the terms of the Creative Commons Attribution 4.0 International license.

10.1128/mSphere.00525-21.3 FIG S3 Correlation analysis between oceanographic variables and proteorhodopsin taxonomic groups. The color gradient indicates the values of Spearman’s correlation coefficient between standardized oceanographic variables. Only values with significance (*P* < 0.05) after Benjamini-Hochberg (BH) false discovery rate (FDR) correction are shown (top panel). Correlation coefficient values for significant correlation are also shown (bottom panel). Download FIG S3, PDF file, 0.6 MB. Copyright © 2021 Cifuentes-Anticevic et al. 2021 Cifuentes-Anticevic et al. https://creativecommons.org/licenses/by/4.0/ This content is distributed under the terms of the Creative Commons Attribution 4.0 International license.

10.1128/mSphere.00525-21.6 TABLE S3 Mean relative abundance of total PR sequences in coastal seawater from Chile Bay by sampling site. Download Table S3, XLSX file, 0.01 MB. Copyright © 2021 Cifuentes-Anticevic et al. 2021 Cifuentes-Anticevic et al. https://creativecommons.org/licenses/by/4.0/ This content is distributed under the terms of the Creative Commons Attribution 4.0 International license.

10.1128/mSphere.00525-21.7 TABLE S4 Mean relative abundance of PR sequence types in coastal seawater from Chile Bay (data from P1 and P3 sampling sites pooled together). Download Table S4, XLSX file, 0.01 MB. Copyright © 2021 Cifuentes-Anticevic et al. 2021 Cifuentes-Anticevic et al. https://creativecommons.org/licenses/by/4.0/ This content is distributed under the terms of the Creative Commons Attribution 4.0 International license.

10.1128/mSphere.00525-21.8 TABLE S5 Permutational multivariate analysis of variance (PERMANOVA) to estimate the impact of the oceanographic variables on the relative abundance of proteorhodopsin-bearing bacteria. Download Table S5, XLSX file, 0.01 MB. Copyright © 2021 Cifuentes-Anticevic et al. 2021 Cifuentes-Anticevic et al. https://creativecommons.org/licenses/by/4.0/ This content is distributed under the terms of the Creative Commons Attribution 4.0 International license.

**FIG 6:**
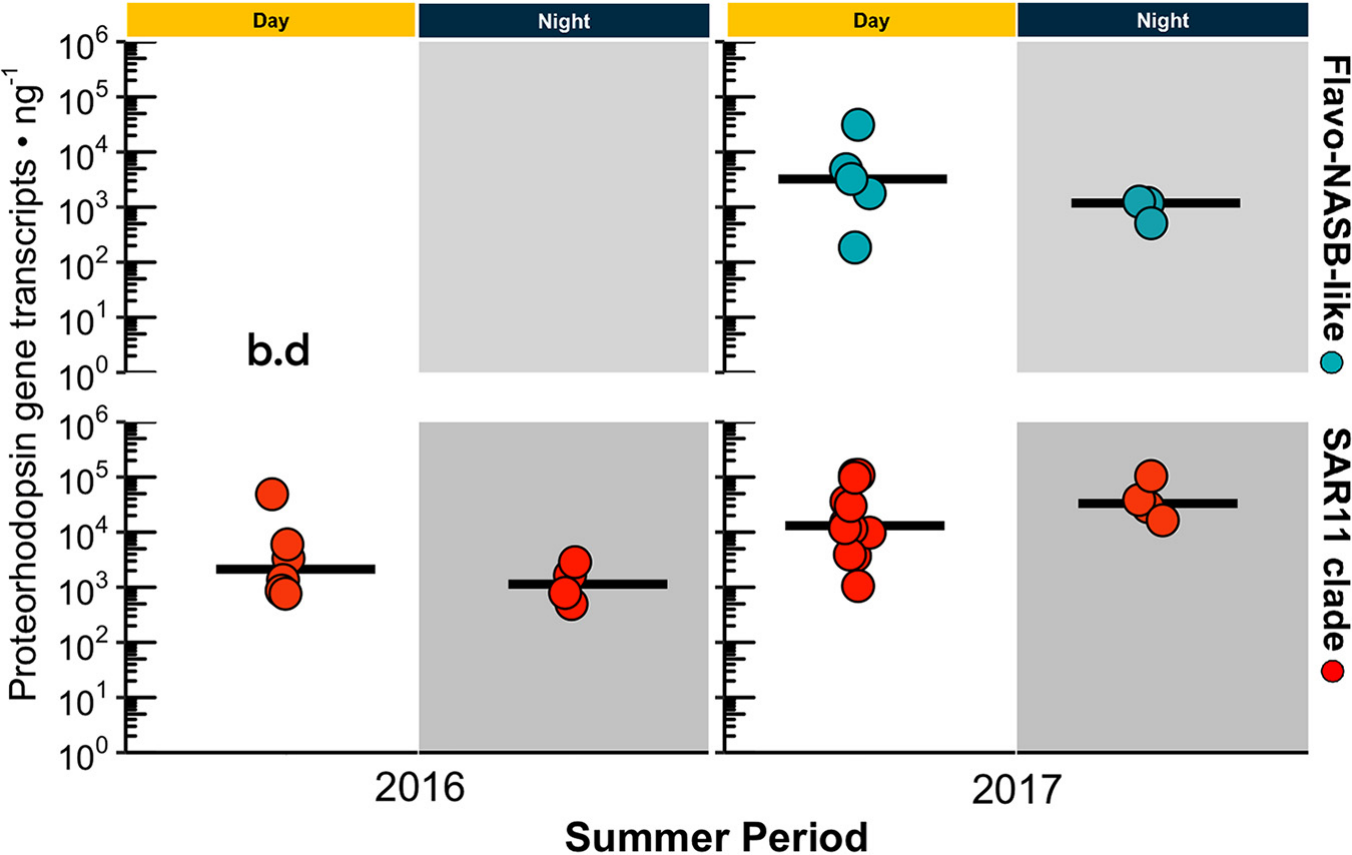
Transcript levels of proteorhodopsin genes of the SAR11 clade and Flavo-NASB-like clade in RNA samples obtained during the day and night in the austral summers of 2016 and 2017. The horizontal line in the graph represents the mean. Flavo-NASB-like proteorhodopsin transcripts were below the detection level (b.d) during the summer of 2016. For 2016, day, *n* = 7; night, *n* = 3. For 2017, day, *n* = 10; night, *n* = 3.

Reverse transcription-qPCR (RT-qPCR) was used to quantify proteorhodopsin gene transcripts from SAR11 and Flavo-NASB-like for daytime and nighttime surface (2 m) samples from 2016 and 2017 to confirm the daily variability of the proteorhodopsin gene transcriptional activity observed in 2014. Results show that proteorhodopsin gene transcription was active during both the daytime and nighttime (Fig. 6). Furthermore, no significant difference in transcription was found between daytime and nighttime for the SAR11 proteorhodopsin gene during both summer periods (Fig. 6). Transcription of the Flavo-NASB-like proteorhodopsin gene in 2016 was below the assay detection limit, while in 2017, no significant difference was found between the day and night (Fig. 6).

## DISCUSSION

### Presence, identity, and spectral tuning of proteorhodopsin in polar waters.

The proteorhodopsin gene’s taxonomic affiliation has been determined for several marine environments (see Table 1 in reference 9). However, proteorhodopsin has received much less attention in polar waters, even though its presence has been detected several times since the first paper on proteorhodopsin (8). Since then, proteorhodopsin genes have been found in Arctic waters (14–16) and either the proteorhodopsin gene or the protein has been reported in Antarctic waters and sea ice (17–19, 26–28). These previous studies used different sampling strategies, seasonality, and identification methods, such as using different primers for retrieving the proteorhodopsin gene. However, some general conclusions can be derived: in both Arctic and Antarctic marine environments, the proteorhodopsin gene is reportedly present during the summer and, unexpectedly, transcribed during winter and nighttime (15, 16, 18, 19). Taxonomic affiliation of Arctic proteorhodopsin gene sequences (15, 16) and those identified in Chile Bay show that the dominant classes were *Gammaproteobacteria*, *Alphaproteobacteria*, and *Bacteroidetes*; however, their relative proportions were different spatially and temporally in the studies for which these patterns could be compared (15, 16). For instance, 2014 Chile Bay waters were dominated by *Bacteroidetes* proteorhodopsin gene sequences, whereas 2016 and 2017 shared the typical higher *Alphaproteobacteria* and *Gammaproteobacteria* proteorhodopsin gene pattern previously reported in the Arctic and other marine regions worldwide (16, 29–31). Despite the fact that the distribution patterns seem to be different in polar regions, proteorhodopsin sequences described in the Arctic (14–16) are phylogenetically placed in the same clades as the proteorhodopsin proteins identified in Antarctica (8, 17). Further analyses should be performed to evaluate whether the proteorhodopsin proteins are adapted to cold or whether the shared sequences of the poles respond to the adaptation of the marine bacteria that carry these proteorhodopsin genes.

The spectral tuning ability of proteorhodopsin has drawn attention since 2001, when Béjà et al. (8) reported blue-PR in the Southern Ocean (Palmer Station) bacterioplankton community. For the Antarctic marine environment, the presence of blue-PR and green-PR has been reported only in sea ice microbial communities from the Ross Sea (17). We retrieved green- and blue-absorbing proteorhodopsins, and even during the high-Chl*a* period (March) reported in Chile Bay 2014 (24), the relative abundance of green- and blue-PR genes did not change from February to March. As blue-PR using organisms might have been outcompeting for the sunlight resource with phototrophic eukaryotes at that period of the year in Antarctica, the green-PR was generally more abundant as they can exploit a different wavelength than Chl*a*-based microorganisms. This result is similar to data from the summer Arctic coastal marine environment (16), thereby confirming the higher relative abundance of green-PR found at high latitude coastal sites (9). Considering that new ice-free coastal areas could appear in the WAP (32), is important to study green-PR photo(hetero)trophy to determine the significance of these microorganisms in these (new) polar coastal areas.

The abundance of proteorhodopsin-bearing bacteria recovered in Chile Bay is at the lower end of the previously reported abundance at diverse environments such as the North Sea (35%) (29), the Chesapeake Bay (40%) (33), the Baltic Sea (46%) (30), the Sargasso Sea (65%) (34), the ultraoligotrophic Eastern Mediterranean Sea, and the San Pedro Channel where the proteorhodopsin-bearing bacteria represented more than 70% of the marine microbial community (35, 36). This low abundance of proteorhodopsin-bearing bacteria in Chile Bay, compared to other nonpolar oceanic regions, may be related to the particular marine community structure or (a)biotic interactions between the proteorhodopsin-bearing bacterial community and the environment. However, the proportion of proteorhodopsin-bearing bacteria has been estimated only in a few studies of polar waters. In the Chukchi and Beaufort Seas, less than 0.4% of the bacterioplankton harbored a proteorhodopsin gene (2 × 10^−3^ proteorhodopsin/16S rRNA) (14). Boeuf et al. (16) estimated that in the Southern Beaufort Sea, 32% of the SAR11 bacteria carried the proteorhodopsin gene and that these accounted 63% of all proteorhodopsin-bearing prokaryotes. Moreover, the number of proteorhodopsin-bearing cells represented up to 45% of the total cells (16). In our 3-year study, proteorhodopsin-bearing bacteria ranged widely from 1.2 to 63.3% of the Antarctic coastal water bacterial community. Although meta-omics showed that *Bacteroidetes* was the main proteorhodopsin-bearing phylum in 2014, the qPCR results suggest a low relative abundance of these bacteria in 2016 and 2017. This may be due to a bias in the primer coverage for *Flavobacteriia* proteorhodopsin gene sequences, designed using North Atlantic and Arctic sequences. However, due to the inability to design universally conserved qPCR primers that cover most currently described proteorhodopsins (16), it is difficult to accurately determine the proteorhodopsin-bearing bacterial composition and abundance through qPCR. Comparison of our results with others from previous reports should be made with caution, due to the different methodologies used in each study, and estimating only the relative abundance of the proteorhodopsin-bearing bacteria that can be retrieved with the qPCR primers used.

### Proteorhodopsin gene expression in polar waters.

Although studies showing the presence of proteorhodopsin gene sequences in polar waters are few, those showing proteorhodopsin gene transcription are substantially less frequent, with the expression of this gene determined in an extremely few cases (17, 19). A study off Palmer Station in Antarctica, which analyzed metaproteomes from six summers and three winters, found only three proteorhodopsin sequences out of 1,061 proteins: two associated with SAR11 and one with the oligotrophic marine *Gammaproteobacteria* group (19). In a seasonal study from early winter to spring in the Amundsen Gulf (Southeastern Beaufort Sea, Arctic Ocean), proteorhodopsin gene transcription was observed at winter darkness in January, after ice breakup in May, and associated with phytoplankton blooms in late June (15). *Gammaproteobacteria* were always the most abundant proteorhodopsin-bearing bacteria, whereas the abundance of proteorhodopsin-bearing *Alphaproteobacteria* and *Bacteroidetes* varied, the former being more abundant in late winter. Koh et al. (17) retrieved only 17 proteorhodopsin sequences from cDNA clone libraries of Ross Sea ice core samples from five locations and three depths. Finally, Boeuf et al. (16) obtained proteorhodopsin sequences from DNA and cDNA along an Arctic coastal transect in the summer, but no quantitative data for proteorhodopsin gene transcription was shown. Thus, our results from Antarctica, which combine metatranscriptomics and RT-qPCR, significantly increase the amount of polar proteorhodopsin gene transcriptional data. Furthermore, the 2014 metatranscriptomic analysis is the first to compare blue- and green-PR transcription levels in polar marine environments, describing that these bacteria use two different sunlight spectra to conserve light energy. Our results show that proteorhodopsin sequences from the WAP exhibited color adaptation, with the blue-PR much more expressed than green-PR, despite its lower abundance. Additionally, SAR11 clade presented mostly blue-PR (but also green-PR at the lower level), whereas *Flavobacteriia* presented (and transcribed) only green-PR.

During the summer of 2014, an interesting pattern emerged regarding the transcriptional activity and the blue/green proteorhodopsin ratio. Green-PR sequences were more abundant in the DNA samples (∼90%), as has been described in the Arctic (16), while blue-PR accounted for about 50% of the transcription. Although most of the green-PR belonged to *Bacteroidetes*, their expression was lower than that of the SAR11 blue-PR. This pattern has been reported only for temperate open ocean waters (ALOHA station) (37), but not for polar marine environments. Although RT-qPCR did not reveal significant differences in proteorhodopsin gene transcription during the day and night between the summers of 2016 and 2017, an overall higher transcription was found during the daytime of summer 2014. Previously, circadian transcriptional activity of the proteorhodopsin gene has been reported only from tropical and subtropical marine environments (38–40). It is also worth noting that the relative abundance of *Flavobacteriia* proteorhodopsin transcripts was lower at night. This suggests a reduction in proteorhodopsin gene transcription at night/dark by *Flavobacteriia* as previously reported (41, 42), resulting in an apparent higher proportion of *Alphaproteobacteria* proteorhodopsin reads.

The (RT-)qPCR results from this study should be taken with caution because the primers used likely did not recover a substantial fraction of the proteorhodopsin diversity found by meta-omics in the 2014 samples. However, some conclusions can be derived. First, the analysis shows large variability in both relative abundance and community composition of proteorhodopsin-bearing bacteria across the three summers. Second, there is a negative correlation between the contribution of SAR11 proteorhodopsin with Chl*a*, which were very different during 2016 and 2017 in Chile Bay at the sampling time. This pattern has been previously described in other polar and temperate oceans (13, 14, 16, 35) and may be related to the oligotrophic lifestyle of proteorhodopsin-bearing SAR11 that benefit from low-molecular-weight dissolved organic matter concentrations (43).

### Ubiquity of proteorhodopsin-bearing bacteria during summer in the WAP.

In the WAP region, aerobic anoxygenic phototrophic bacteria account for up to 8% of the community (7), while photosynthetic cyanobacteria are a minor component of the marine community (19, 44–46). Thus, photoautotrophy in this system mostly relies on eukaryotic microorganisms, whose activity increases during blooms, like those described during the austral summer in Chile Bay (24, 25). Our results demonstrate that every late summer and early autumn in Chile Bay, proteorhodopsin-bearing bacteria are a ubiquitous and dominant group of phototrophic microorganisms, and this may be extrapolated to the WAP coastal waters. Here, we demonstrate not only the presence but also the transcriptional activity of proteorhodopsin-bearing bacteria across three different austral late summers, and we identified that differences in their abundance may be related to environmental parameters. The relative abundance of SAR11 proteorhodopsin negatively correlates with Chl*a* levels, while SAR92 proteorhodopsin positively correlates with phosphate levels, as previously reported for the Arctic (16). The negative correlation between the SAR11 clade proteorhodopsin abundance and Chl*a* concentrations has also been previously reported for the North Atlantic Ocean (14, 47).

It becomes necessary to determine how these environmental variables drive proteorhodopsin-bearing bacterial composition, abundance, and activity in Antarctic waters to model proteorhodopsin-bearing bacterial dynamics over the incoming years. Further ecological and biochemical studies will also be required to fully understand how proteorhodopsin contributes to microbial energetic metabolism and how light availability might influence or affect rhodopsin photoheterotrophy, particularly in rapidly changing environments such as Antarctica. This is especially important under the present climate change scenario because as some environmental variables change in the ocean (48), it will be relevant to predict how sunlight energy will be used by marine bacteria in the Southern Ocean.

## MATERIALS AND METHODS

### Sampling site.

Seawater samples were collected from Chile Bay on Greenwich Island, South Shetland Island, Antarctica (Fig. 1). Samples for meta-omics were collected during 2014 (February and March) (24). Samples for quantitative PCR (qPCR) were collected as a time series during the late summer of 2016 (18 February to 4 March 2016) and 2017 (8 February to 25 February 2017) (see Table S1 in the supplemental material). Surface (2 m) and subsurface (30 m) seawater was sampled during the day and night at two locations in Chile Bay: (i) P1, which was close to the “Fuerza Aérea” glacier (62°29′2′′ S – 59°40′6′′ W); and (ii) P3, which was more exposed to the open ocean (62°27′6′′ S – 59°40′6′′ W). In the summer of 2016, on February 18, sunrise at 05:30 h and sunset at 20:53 h, while for March 4, the sunrise and sunset at 6:14 h and 20:04 h, respectively. During the summer of 2017, sunrise/sunset was at 05:01/21:22 h and 05:53/20:28 h, at the beginning and end of this period, respectively. From a small inflatable boat, seawater was first collected using a hand-operated membrane pump, deposited into clear 20-liter acid-washed (HCl [10%]) bottles, and then transported to the INACH (Instituto Antártico Chileno) laboratory at Chile Bay for processing.

### Environmental variables.

Seawater temperature (°C), salinity, and oxygen (milliliter liter^−1^) at the P1 and P3 sampling locations were obtained using a CTD (conductivity, temperature, depth) profiling sensor (Seabird 19; Sea-Bird Electronics, Bellevue, WA, USA). Salinity, oxygen, and ammonium (NH_4_^+^) were measured only for the 2017 samples. To determine nutrients, namely, nitrite, nitrate, phosphate, and silicic acid, triplicate prefiltered (0.7-μm GF/F glass fiber filter) seawater samples were collected in 15-ml polyethylene flasks at P1 (2-m depth) and P3 (2-m and 30-m depth) and stored at –20°C until further analysis. Nutrient concentrations were determined using standard colorimetric techniques with a segmented flow Seal AutoAnalyzer3 (SEAL Analytical GmbH, Norderstedt, Germany) at Universidad de Concepcion as described previously (24). NH_4_^+^ was measured as previously described (49). For each location and depth, Chl*a* was determined in triplicate by filtering 1 liter of seawater through 0.7-μm GF/F glass fiber filters, which were frozen at –20°C until laboratory analyses by acetone extraction and fluorometric measurements (50).

### DNA and RNA extraction.

For DNA and RNA analysis of the 2016 and 2017 samples, microbial biomass from 3 to 4 liters of seawater was prefiltered through 200-μm nylon mesh followed by a 20-μm polycarbonate filter using a peristaltic pump (6 to 600 rpm) (model no. 7553–70; Cole Parmer, Vernon Hills, IL, USA) at 50 to 100 ml min^−1^. The subsequent filtrate was concentrated onto 0.22-μm-pore-size Sterivex units (Millipore, Burlington, MA, USA). The filters for RNA analysis were preserved in RNAlater (Invitrogen, Carlsbad, CA, USA). Both RNA and DNA filters were maintained at –80°C until laboratory processing at the Pontifical Catholic University of Chile, Santiago, Chile. DNA was extracted according to Tillett and Neilan (51) with modifications. Briefly, filters were resuspended in lysis buffer (1% potassium ethyl xanthogenate [Sigma-Aldrich, St. Louis, MO, USA], 100 mM Tris-HCl [pH 7.4], 20 mM EDTA [pH 8], 800 mM ammonium acetate). Sterile glass beads were added and then shaken in a BeadBeater for 30 s. Next, the mixture was incubated with sodium dodecyl sulfate (SDS) (1% final concentration) at 65°C for 2 h and then placed on ice for 30 min. DNA was extracted with phenol-chloroform-isoamyl alcohol (25:24:1), and the residual phenol was eliminated with chloroform-isoamyl alcohol (24:1). The extract was cleaned by overnight precipitation with cold isopropanol and then washed with 70% ethanol. DNA was quantified using the Qubit 2.0 fluorometer (Thermo Fisher Scientific, MA, USA), the quality (*A*_260_/*A*_280_) was assessed spectrophotometrically, and the integrity was checked by agarose gel electrophoresis. RNA was extracted from the filters using TRIzol (Invitrogen) and the RNA Clean & Concentrator kit (Zymo Research, USA). To eliminate any remaining DNA, 1 μg of RNA from each sample was treated with DNase (Turbo DNase; Invitrogen), and the absence of DNA contamination was assessed analyzing the 16S rRNA gene by PCR using the primers and conditions described below.

### Identification of proteorhodopsin gene sequences from 2014 meta-omes.

Identification of proteorhodopsin protein-encoding genes and proteorhodopsin gene transcription analysis were performed using previously obtained sequence data from the 0.22- to 20-μm bacterial fraction of the 2014 samples (24). Briefly, surface seawater samples were collected in 2014 on February 11 (nighttime, 21:00 h [local time UTC-3]) and 14 (daytime, 11:00 h) and March 3 (daytime, 13:30 h) and 4 (nighttime, 21:00 h) (Table 2). DNA and RNA were extracted and processed as previously described (24). The metagenomic and metatranscriptomic sequencing data from the 0.22- to 20-μm bacterial fractions used in this study are available at NCBI under BioProject accession no. PRJNA421008. Quality trimming of metagenomic reads was performed using Prinseq (83): a hard clipping of the first 7 leftmost bases and 9 leftmost bases for February and March metagenomes, respectively, mean read quality of 30, and 3′ trimming for bases with quality below 30. Similarly, for the metatranscriptomes, a minimum quality of 30, a minimum length of 30 bp, and a hard clipping of the first 11 bases was performed. Low complexity sequences and undetermined bases were filtered (-ns_max_p 0 -lc_method dust -lc_threshold 7) (83) as described previously (52). Trimmed reads were assembled with SPAdes software v3.10.1 (meta option) (53). Contigs larger than 500 bp were used for protein and gene prediction via Prodigal v2.6.3 with meta mode and bypassing the Shine-Dalgarno sequence (54). Identification of proteorhodopsin candidates was made with predicted proteins from both metagenomes using DIAMOND (BLASTP; E value ≤ 10^−7^) (55) against the curated database MicRhoDE (56), particularly with those annotated as proteorhodopsin. Next, the potential proteorhodpsins were evaluated using the Pfam database (57) (HMMER 3.0; http://hmmer.org/), identifying the HMM profile Bac_rhodopsin (PF01036; hmmscan; trusted cutoff) and selecting those belonging to the InterPro family proteorhodopsin (IPR017402) when classified with InterProScan (58). The resulting proteins were manually curated, identifying the presence of the proton pumping functional domain at the C-helix and those larger than 100 amino acids.

### Abundance and expression of the proteorhodopsin gene in the 2014 meta-omes.

To determine the relative abundance of proteorhodopsin in the 2014 metagenomes, Bowtie2 v2.2.6 (59) (sensitive; default) was used to recruit reads from the metagenome to the corresponding proteorhodopsin gene. Next, single-copy housekeeping genes *recA*, *rplB*, *rpoB*, and EF-Tu were identified in the predicted proteins of the metagenomes using HMMSearch with the HMM profiles PF00154, PF00181, PF04563, and PF00009, respectively, and then they were verified as belonging to the InterPro families IPR013765, IPR002171, IPR015712, and IPR004541, respectively (33, 34, 36). The average normalized abundance of the proteorhodopsin gene in the metagenomic sequence data was calculated as the average of each ratio of the proteorhodopsin gene to single-copy housekeeping gene, where the number of reads recruited to each gene was normalized by the recruited gene length. To estimate proteorhodopsin gene transcription, we analyzed the RNA sequencing data. Quality trimming of metatranscriptomic reads was performed as described previously (52), and the remaining rRNA sequences were removed using SortMeRNA (60) (default parameters). Nonaligning reads were mapped with Bowtie2 (59) (sensitive; default) to the set of proteorhodopsin genes. Enzymes of the retinal biosynthetic pathway, namely, 15,15′-β-carotene dioxygenase (*blh*), phytoene synthase (*crtB*), and lycopene cyclase (*crtY*), were identified and quantified in the same manner as the single-copy housekeeping genes, but using the HMM profiles PF15461, PF00494, and PF05834, respectively (31). To assess whether there was a difference in the transcription levels between the four metatranscriptomes, we analyzed the *rplB*, *rpoB*, and EF-Tu genes. Single-copy housekeeping gene reads from the four metatranscriptomes were obtained by the same procedure described above. The relative abundance in each metatranscriptome of the proteorhodopsin gene and retinal biosynthetic pathway enzymes was expressed as the transcript abundance of each gene times the average abundance of the housekeeping genes. Although the *recA* gene has been used as a housekeeping gene to normalize the expression of functional genes in metatranscriptomes (61), it apparently does not exhibit constitutive levels of expression in Antarctic marine waters (62, 63). Therefore, we used *recA* only to normalize proteorhodopsin gene abundance in metagenomes but not to normalize transcriptional activity.

### Phylogenetic analysis of proteorhodopsin sequences.

A reference tree was built with the retrieved proteorhodopsin proteins from the metagenomes and the “strain_only=strain” sequences from the MicRhoDE database (56). The protein sequences from Chile Bay and the MicRhoDE database were first clustered separately using cd-hit (64, 65) to 82% identity (16) and then aligned with MAFFT (G-INS-i) (66). The multiple sequence alignment was trimmed using trimAl (-gt 0.2) (67), and then the tree was reconstructed with IQ-TREE (automatic model detection, -bb 10000 -alrt 10000) (68, 69). Short amplicon protein sequences from polar marine environments (8, 14–17) were phylogenetically placed using EPA-ng algorithm (70) to the reference proteorhodospsin tree. The resulting tree was processed with GAPPA (71) and then visualized in iTOL (72). To determine the proton pumping amino acids at positions 97, 101, and 108 and the spectral tuning switch amino acid at position 105, the protein sequences were screened using the multiple sequence alignment with the positions previously identified in the references sequences and visualized in Skylign (73).

### Quantification of proteorhodopsin gene abundances and transcription.

qPCR was used to determine the abundances of three proteorhodopsin gene types and the bacterial 16S rRNA gene in the 2016 and 2017 DNA samples using the primers listed in Table S3. In this study, specific primers were used to target the proteorhodopsin gene of *Flavobacteriia* from the NASB clade (47), *Alphaproteobacteria* (clade SAR11) (42), and *Gammaproteobacteria* (clade SAR92) (16), and universal bacterial 16S rRNA gene primers (74). To obtain the standard curve (10^8^ to 10^2^ copies) for proteorhodopsin gene quantification, the three proteorhodopsin gene types were cloned into the pGEM-T Easy Vector (Promega, Madison, WI, USA). Plasmid DNA was linearized with SacI (Thermo Fisher) and quantified using NanoDrop (Thermo Fisher Scientific). Standard curves for the 16S rRNA gene were generated by amplifying Escherichia coli DNA and then purifying the amplicon with GeneJET Gel Extraction kit (Thermo Fisher Scientific). qPCRs were performed in triplicate using 1 μl of DNA (1 ng μl^−1^) in a final volume of 15 μl with the SensiMix kit (Bioline GmbH, Luckenwalde, Germany) and a LightCycler 480 (Roche Holding AG, Basel, Switzerland) real-time qPCR device. The program was as follows: (i) 95° for 10 min and (ii) 40 cycles, with 1 cycle consisting of 10 s at 95°C, annealing for 20 s at the primer-specific temperature (Table S6), and 30 s at 72°C. Proteorhodopsin gene copy numbers were normalized to the 16S rRNA gene copy numbers, assuming 1.9 copies of 16S rRNA and 1 copy of proteorhodopsin gene per genome (16, 34). cDNA was synthesized from 750 ng of DNA-free RNA using the iScript Select cDNA Synthesis kit (Bio-Rad, Hercules, CA, USA) with the same SAR11 and *Flavobacteriia* primers used for the qPCR. Quantification was performed as described above for the DNA samples.

10.1128/mSphere.00525-21.9 TABLE S6 Primers used for qPCR analysis (proteothodopsin gene and bacterial 16S rRNA). Download Table S6, XLSX file, 0.01 MB. Copyright © 2021 Cifuentes-Anticevic et al. 2021 Cifuentes-Anticevic et al. https://creativecommons.org/licenses/by/4.0/ This content is distributed under the terms of the Creative Commons Attribution 4.0 International license.

### Statistics.

The conducted statistical analyses were performed in R with the stats (75), vegan (76), hmisc (77), and corrplot (78) packages. Oceanographic variables were standardized using the z-score method (mean 0, variance 1). Missing values in the temperature factor were computed by linear interpolation with the imputets (79) package. Spearman’s rank correlation (rho) (80) was performed to estimate simple correlations (*P* < 0.05) between standardized oceanographic factors (i.e., physicochemical and biological) (*N* = [2016] day = 7, night = 3; [2017] day = 15, night = 4). For multiple comparisons, adjusted *P* values were obtained using the Stats package and Benjamini-Hochberg (BH) *post hoc* tests (81). To estimate the variance of taxonomic group proteorhodopsin percentage abundances by sampling dates, depths, and between taxonomic groups, the Kruskal-Wallis rank sum analysis was applied (81) (df = 2, *n* = *N*). A permutational analysis of variance (PERMANOVA) (82) with marginal effect was applied to taxonomic group as the explanatory factor (df = 1, *n* = *N*) using the function adonis2 of the R vegan package (9,999 permutations) to estimate the impact of the oceanographic variables on the proteorhodopsin percentage abundances.

### Data availability.

Raw metagenomic data for each metagenome and metatranscriptome were deposited in the Sequence Read Archive database under BioProject accession number PRJNA421008.
