# Supplementary figures and images for: Proteorhodopsin Phototrophy in Antarctic Coastal Waters
Source: mSphere. 2021 Aug 18;6(4):e00525-21. doi: 10.1128/mSphere.00525-21 (PMC8386455; doi:10.1128/mSphere.00525-21)

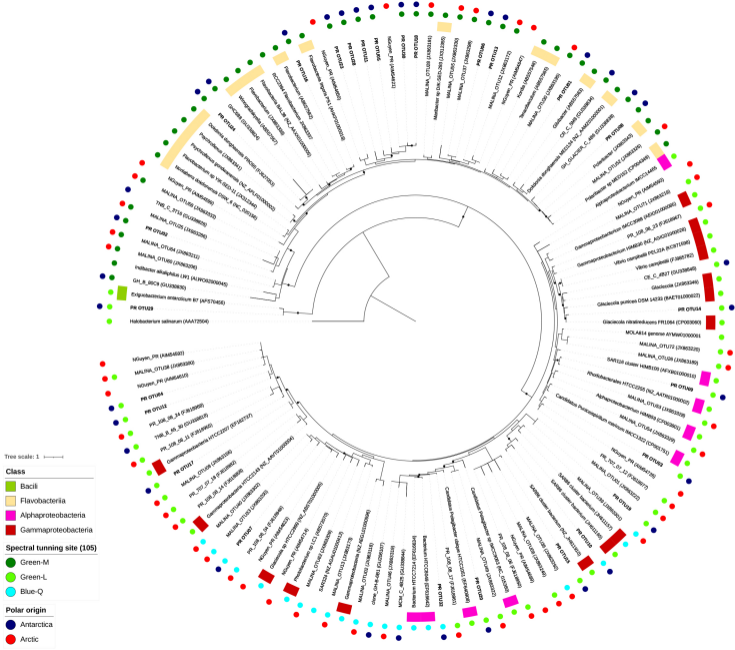

Supplement: FIG S1 [file msphere.00525-21-sf001.pdf]

Flavobacteriia-NASB-like SAR92 clade (MALINA OTU11) SAR11 clade

2 m

30 m

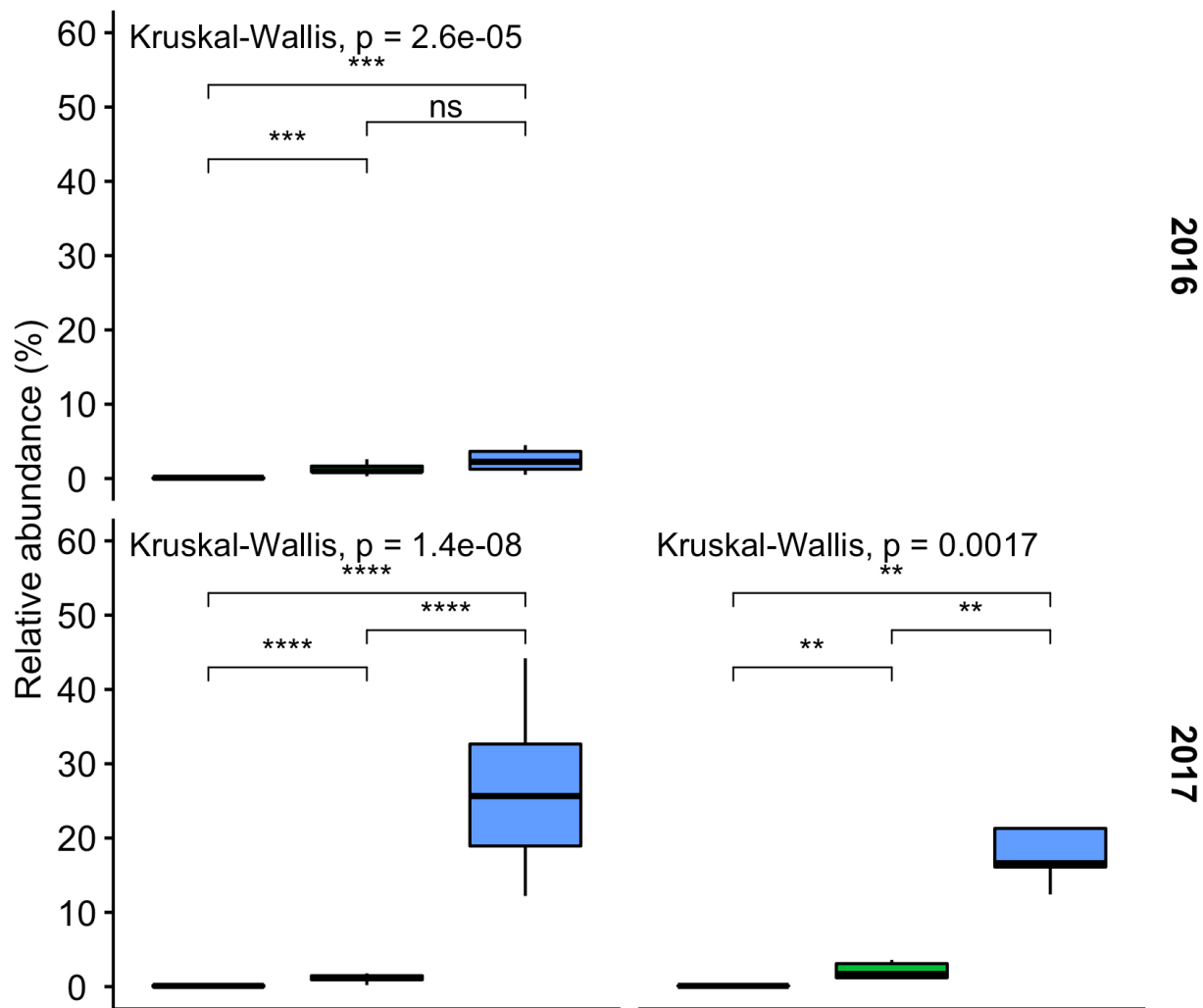

Supplement: FIG S2 [file msphere.00525-21-sf002.pdf]

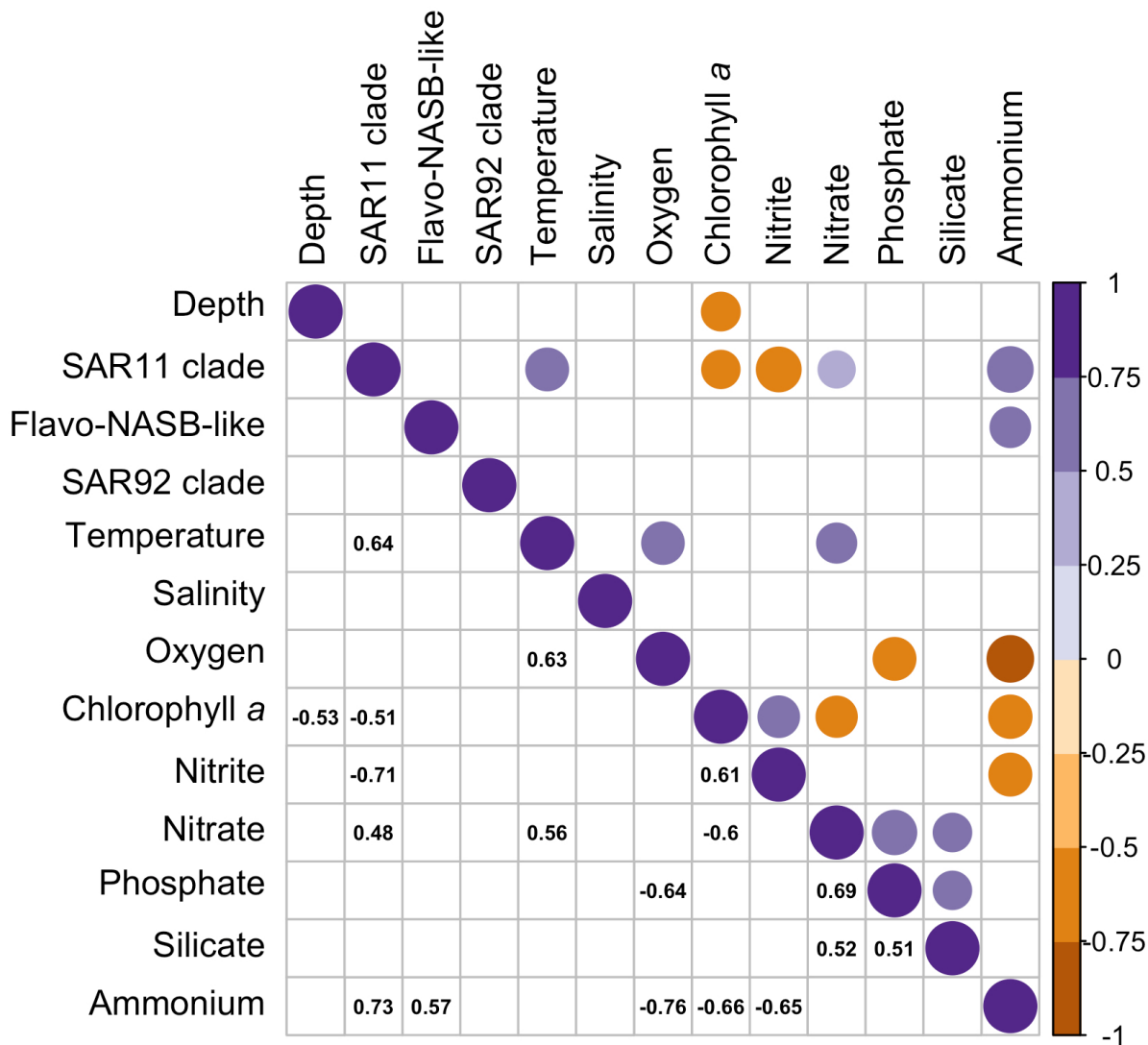

Supplement: FIG S3 [file msphere.00525-21-sf003.pdf]
